# An octave-spanning mid-infrared frequency comb generated in a silicon nanophotonic wire waveguide

**DOI:** 10.1038/ncomms7310

**Published:** 2015-02-20

**Authors:** Bart Kuyken, Takuro Ideguchi, Simon Holzner, Ming Yan, Theodor W. Hänsch, Joris Van Campenhout, Peter Verheyen, Stéphane Coen, Francois Leo, Roel Baets, Gunther Roelkens, Nathalie Picqué

**Affiliations:** 1 Photonics Research Group, Department of Information Technology, Ghent University–imec, Sint-Pietersnieuwstraat 41, 9000 Ghent, Belgium; 2 Center for Nano- and Biophotonics (NB-Photonics), Ghent University, 9000 Ghent, Belgium; 3 Max Planck Institut für Quantenoptik, Hans-Kopfermannstrasse 1, 85748 Garching, Germany; 4 Ludwig-Maximilians-Universität München, Fakultät für Physik, Schellingstrasse 4/III, 80799 Munich, Germany; 5 imec, Kapeldreef 75, 3001 Leuven, Belgium; 6 Department of Physics, The University of Auckland, Private Bag 92019 Auckland, New Zealand; 7 Institut des Sciences Moléculaires d’Orsay, CNRS, Bâtiment 350, 91405 Orsay, France

## Abstract

Laser frequency combs, sources with a spectrum consisting of hundred thousands evenly spaced narrow lines, have an exhilarating potential for new approaches to molecular spectroscopy and sensing in the mid-infrared region. The generation of such broadband coherent sources is presently under active exploration. Technical challenges have slowed down such developments. Identifying a versatile highly nonlinear medium for significantly broadening a mid-infrared comb spectrum remains challenging. Here we take a different approach to spectral broadening of mid-infrared frequency combs and investigate CMOS-compatible highly nonlinear dispersion-engineered silicon nanophotonic waveguides on a silicon-on-insulator chip. We record octave-spanning (1,500–3,300 nm) spectra with a coupled input pulse energy as low as 16 pJ. We demonstrate phase-coherent comb spectra broadened on a room-temperature-operating CMOS-compatible chip.

## Abstract (web summary)

Phase-coherent frequency combs in the mid-infrared have important potential applications but their fabrication remains challenging. Here, Kuyken *et al*. demonstrate an octave-spanning frequency comb in the mid-infrared using a highly nonlinear dispersion-engineered silicon waveguide on a silicon-on-insulator chip.

## Graphical abstract

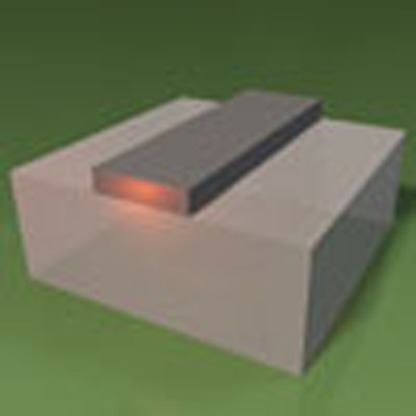

Frequency combs in the mid-infrared region^1^ have been mostly generated by nonlinear frequency conversion of near-infrared frequency combs. Although the field is currently very active with the exploration of many different and promising approaches^2^,^3^,^4^, producing a very broad spectrum with a slowly varying envelope remains challenging. Supercontinuum generation in a highly nonlinear fibre is known, under certain circumstances^5^, to be a powerful way to generate an octave-spanning frequency comb.

However, in the mid-infrared spectral region, suitable materials have remained scarse and difficult to engineer. Phase-coherent octave-spanning frequency comb generation has been achieved by spectral broadening of optical parametric oscillators (OPOs)^6^ and thulium-doped fibre laser^7^,^8^,^9^ frequency combs in nonlinear chalcogenide-tapered fibres. Taper lifetimes have recently been improved to several days with hybrid silica-chalcogenide structures, in which octave-spanning frequency comb generation has been reported^8^,^9^ using 65-fs pulses of only 18 pJ. Promising solutions for enhanced stability are presently under investigation with multimaterial chalcogenide nanotapers^10^. Another approach is the use of quasi-phase-matched periodically poled lithium niobate (PPLN) waveguides. Impressive results have been obtained and an octave-spanning phase-coherent supercontinuum has been generated^11^. However, absorption between 2,500 and 2,800 nm, and more importantly the limited transparency of lithium niobate beyond 4,500 nm, inhibits the scaling of the technology to longer wavelengths. Furthermore, high-energy pulses (7 nJ) are needed because of the moderate nonlinearity of the waveguide. In addition, during the poling of the crystal small random variations on the location of the walls of the poled domains are introduced. This aberration increases the conversion of parasitic processes significantly^11^,^12^ and makes modelling difficult.

Silicon-based waveguides have been originally conceived for the telecommunication region. In this region, octave-spanning supercontinuum generation has been demonstrated by pumping silicon nitride waveguides with 150-pJ pulses centred at 1.3 μm (ref. 13), but the coherence conservation in the supercontinuum generation process has not been investigated. Recently, the application of silicon technology to the mid-infrared spectral region has attracted significant interest^14^,^15^,^16^. Silicon nanophotonic wire waveguides can be engineered^17^ within a nanometre precision in a standard CMOS facility. Such waveguides offer many advantages for mid-infrared nonlinear optics, mostly related to the wide transparency range of silicon (1.1–8 μm), its high nonlinear refractive index, the possibility of precise dispersion engineering of the waveguide platforms and the high refractive index contrast between the silicon waveguide core and the cladding material (typically, SiO_2_ or air), which allows for densely integrated waveguide systems with a nonlinear parameter one to two orders of magnitude higher than that possible in the chalcogenide or silicon nitride systems.

In this article, we report on the design of strongly nonlinear, dispersion-controlled silicon photonic wire waveguides. We harness such chemically stable waveguides for mid-infrared supercontinuum generation, and we demonstrate a phase-coherent frequency comb generator with a 30-dB bandwidth spanning from 1,540 up to 3,200 nm, with coupled input pulse energies as low as 16 pJ.

## Results

### A highly nonlinear dispersion-engineered silicon waveguide

**Figure 1:**
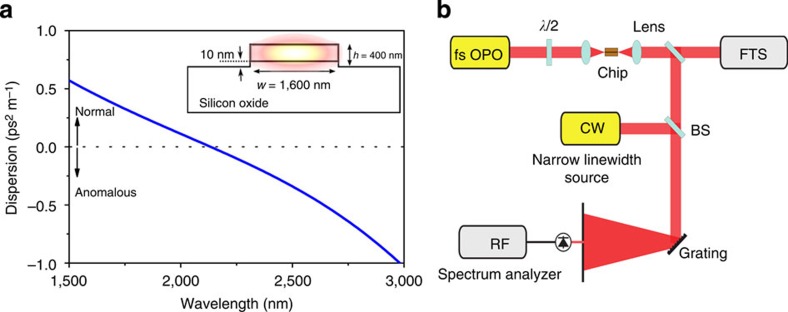
The simulated dispersion of the photonic wire waveguide and the experimental set-up. (**a**) The zero-dispersion wavelength of the quasi-TE mode is at 2,180 nm, while the dispersion is normal at shorter wavelengths and anomalous at longer wavelengths. The waveguide cross-section is shown in the inset. (**b**) Experimental set-up: the OPO pumped by a Ti-Sapphire mode-locked laser is coupled to the silicon chip with a lens. The output of the chip can be sent to a photodetector or a spectrometer.

The photonic wire is fabricated in a CMOS pilot line^17^ on a 200-mm silicon-on-insulator (SOI) wafer and consisting of a 390-nm-thick silicon device layer on top of a 2-μm buried oxide layer. The inset in Fig. 1a) shows a schematic cross-section of the silicon photonic wire. The 1-cm-long air-clad photonic wire has a rectangular cross-section of 1,600 nm × 390 nm. The waveguide is slightly over etched by 10 nm into the buried oxide. The photonic wire widens near the cleaved facets to a 3-μm wide waveguide section for improved coupling efficiency. As a result of the high nonlinear index of silicon^18^ and the strong optical confinement obtained by the high linear refractive index of silicon, the nonlinear parameter in the silicon wire is 38 (Wm)^−1^ at 2,300 nm for the highly confined quasi-TE mode. Such a high nonlinear parameter in silicon waveguides shows the advantage of using silicon over chalcogenide tapers (*γ*=4.5 (Wm)^−1^ (ref. 6)) and silicon nitride waveguides (*γ*=1.2 (Wm)^−1^ (ref. 13)) where the nonlinear parameter is much lower. As a result of the high confinement, the waveguide dispersion of the silicon photonic wire contributes strongly to the overall dispersion of the optical waveguide, such that group velocity dispersion can be engineered by optimizing the waveguide dimensions. The group velocity dispersion of the quasi-TE mode of the dispersion-engineered photonic wire waveguide as a function of wavelength is shown in Fig. 1a. The group velocity dispersion is simulated with the help of a finite element mode solver (Fimmwave). The zero-dispersion wavelength is at 2,180 nm and the dispersion becomes positive (normal) at shorter wavelengths, while the dispersion remains low over a wide spectral band. By using a cut-back technique the propagation loss for the quasi-TE mode is determined to be <0.2 dB cm^−1^ in the wavelength range of 2,200–2,400 nm.

### The experimental set-up for supercontinuum generation

The set-up is shown in Fig. 1b. The frequency comb seed source consists of a homemade mid-infrared singly resonant OPO^19^ at a repetition frequency of 100 MHz, synchronously pumped by a femtosecond mode-locked Ti-Sapphire laser. The OPO is tuned to a centre wavelength of 2,290 nm, close to the zero-dispersion wavelength of 2,180 nm of the silicon waveguide. Pumping a waveguide close to the zero-dispersion wavelength in the anomalous region allows for broadband supercontinuum generation^5^. The OPO has a pulse duration of 70 fs (see Supplementary Fig. 1), while its average power is 35 mW. The ultrashort mid-infrared fs pulses coming from the OPO are coupled to the quasi-TE mode of the silicon photonic wire using a high (Numerical Aperture) NA (NA=0.85) chalcogenide lens with a focal length of 1.87 mm (see Supplementary Methods for details). The output of the chip is coupled, using another chalcogenide lens, to a Fourier transform spectrometer to quantify the spectrum of the output pulses. The coupling loss at the input waveguide facet is estimated to be 12 dB, leading to an on-chip peak power of 225 W or pulse energy of 16 pJ. The high coupling loss at the waveguide facet stems from the bad overlap of the quasi-TE mode of the waveguide and the mode profile at the focus plane of the lens. However, spot size converters^20^ could be used to significantly improve the coupling efficiency. We note that the coupled pulse energy and pulse duration that we use are similar to that used in ref. 8 for phase-coherent supercontinuum generation in a chalcogenide-silica hybrid waveguide.

### Spectral broadening in a silicon photonic nanowire waveguide

**Figure 2:**
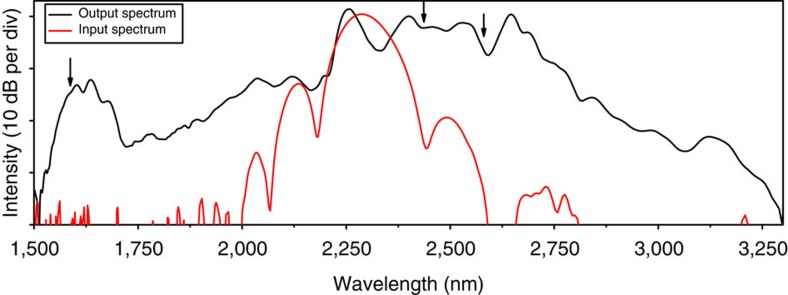
The spectrum at the input (red) and the output (black) of the silicon nanowire. The input pulses are centred at 2,290 nm and have a coupled peak power of 225 W. Their spectrum is broadened in the silicon photonic wire such that it experimentally spans more than an octave: the 30-dB bandwidth covers from 1,540 to 3,200 nm. The arrows indicate the wavelength position where the phase coherence measurements are performed.

The spectra at the input and output of the waveguide are shown in Fig. 2) for a pulse energy of 16 pJ. Spectra at lower pulse energies can be found in Supplementary Fig. 2. The spectrum of the pulses is significantly broadened in the silicon photonic wire waveguide and spans more than an octave: the 30-dB bandwidth spans from 1,540 up to 3,200 nm at the output. The peak at 1,600 nm is located in the normal dispersion regime of the waveguide and is generated through dispersive wave generation, a method used to spectrally extend a supercontinuum^16^. In the course of the several weeks of experimental investigations, we did not observe any modification of the characteristics of the supercontinuum at the output of the silicon waveguide. Consistently, SOI platforms are used in electronics^21^ and optics^22^ during years without degradation.

### A phase-coherent supercontinuum

**Figure 3:**
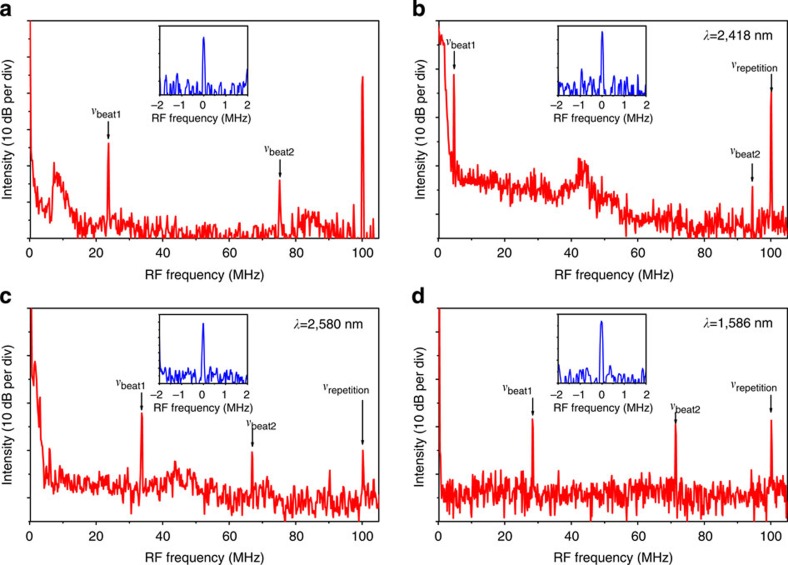
Experimental radiofrequency spectra of the beat notes. (**a**) Radiofrequency spectrum of the free-running beat note of the pump pulses and a narrow line-width source at 2,400 nm. (**b**–**d**) Free-running beat notes of the spectrally broadened pulses and a narrow line-width source at *λ*=2,418 nm, *λ*=2,580 nm and *λ*=1,586 nm, respectively. The insets in the figure show a high-resolution spectrum of the free-running beat notes. The free-running beat notes of the output pulses are measured to be ~50 kHz wide and are not broadened as compared with beat notes measured on the input pulses.

We experimentally investigate the phase coherence of the supercontinuum generated in the waveguide by beat note measurements with a set of narrow line-width continuous-wave lasers. Such a characterization technique for assessing comb coherence properties is an alternative to that involving a *f*–2*f* interferometer^23^ and it is well documented in the literature, for example refs 3, 4, 24. Here it was chosen because our foreseen applications^25^ to molecular spectroscopy do not require self-referencing of the comb. In this characterization, all laser systems, including the continuous wave ones, are free-running. First, we beat the free-running seed source with a tunable continuous wave OPO (Argos Aculight, line-width ~60 kHz at 500 μs) at 2,400 nm on a fast InGaAsSb photodetector (Fig. 3a). We then beat the supercontinuum output with the same OPO (Fig. 3b,c), respectively, tuned at 2,418 and 2,580 nm. We finally beat (Fig. 3d), on a fast InGaAs detector, the supercontinuum with a narrow line-width erbium-doped fibre laser (Koheras AdjustiK E15, NKT Photonics, line-width 0.1 kHz at 100 μs) at 1,586 nm, far from the seed wavelength. All radiofrequency spectra are recorded with a 100-kHz resolution bandwidth, and a spectrum with a 105-MHz span shows three isolated lines. The strong beat signal at 100 MHz corresponds to the repetition frequency of the fs OPO, while the other two beat notes correspond to the beat signal generated by the continuous wave lasers and the two spectrally closest lines of the frequency comb. The line-width of the beat notes, measured with a 10-kHz-resolution bandwidth (insets in Fig. 3a–d) is limited by the instabilities of the free-running lasers, but it is found to be ~50 kHz, without noticeable broadening relative to the fs OPO seed source. The width of the free-running beat notes is the convolution of the width of the two beating laser lines. However, the width of the lines of the free-running femtosecond mode-locked Ti-Sapphire laser used to synchronously pump the seed fs OPO is similar. Stabilizing the system against a radiofrequency reference, such as a caesium clock, is not expected to bring significant line-width reduction: the locking electronics would need a bandwidth that only compensates for slow fluctuations (~100 Hz) to avoid ‘coherence collapse’ by multiplication of the phase noise of the radiofrequency reference^26^. We note that our measured line-widths are in full agreement with that of other free-running or radiofrequency-referenced frequency comb systems^26^. Our investigation thus demonstrates the frequency comb structure of the supercontinuum.

### Comparison with simulations

**Figure 4:**
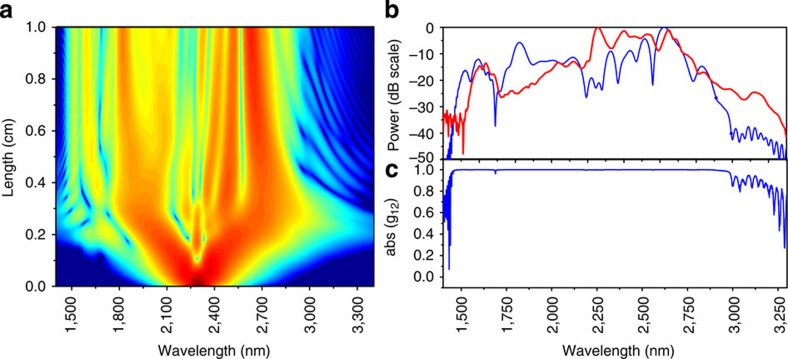
The simulated spectral broadening and coherence of the pulses. (**a**) Evolution of the spectral content of the optical pulse along the length of the silicon photonic wire waveguide. (**b**) Simulated spectra after 1 cm of propagation in the waveguide (blue) and the measured supercontinuum (red). (**c**) Simulated coherence as a function of wavelength.

The coherence of the supercontinuum can be simulated and such simulations can be used to confirm the frequency comb structure at the probed wavelengths as well as indicating the coherence over the whole bandwidth. The supercontinuum generation can be simulated by solving the generalized nonlinear Schrödinger equation numerically with a split-step Fourier method^5^ (see Methods). The simulation takes the linear propagation loss, the nonlinear phase shift, the three-photon absorption and both the induced absorption and dispersion by the carriers into account. In the simulation the nonlinear parameter *γ* is assumed to be 38 (Wm)^−1^, the linear propagation loss is assumed to be 0.1 dB cm^−1^ and the three-photon absorption coefficient is assumed to be 0.025 cm^3 ^GW^−2^ (ref. 27). Figure 4a) shows the evolution of the spectrum of a 225-W peak power, 70-fs-long pulse as it is propagating along the silicon photonic wire waveguide. The simulated spectrum after 1-cm propagation is shown in Fig. 4b. As shown in Fig. 4b, the simulation agrees very well with the experimental results. The simulation of the spectral evolution of the pulse along the photonic wire length reveals (Fig. 4a) that, in the first millimeter of propagation, self-phase modulation is the primary mechanism for spectral broadening. The spectrum is further broadened into the telecom wavelength range, where the group velocity dispersion of the waveguide is normal, through dispersive wave generation^28^. The use of the short pulses favours the processes such as dispersive wave generation and self-phase modulation. Unlike in ref. 29 where longer, ps pulses were used and the spectral broadening primarily results of amplification of background noise (modulation instability), the nonlinear process of dispersive wave generation and self-phase modulation maintain the coherence in the pulse.

The coherence of the supercontinuum can be simulated by including shot noise at the input. The noise *E*_noise_(*t*) at the input is assumed to be a random variable with a stochastic distribution 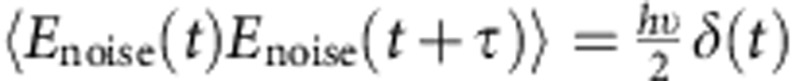, with *h* the Planck constant and *υ* the frequency of the photons, and analysing an ensemble of simulated supercontina^30^. The first-order coherence function


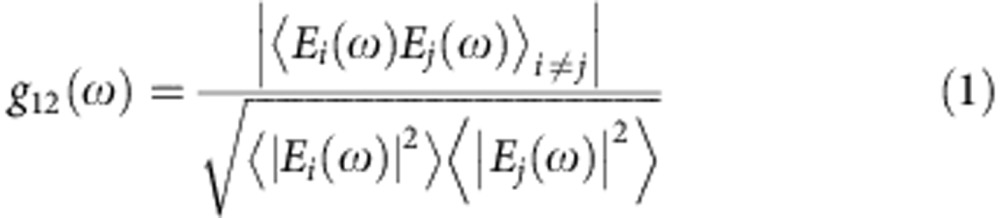


is calculated for an ensemble of 100 spectra and is shown in Fig. 4c. The coherence is close to unity over the whole spectrum, indicating that the generated supercontinuum is coherent over its entire bandwidth.

**Figure 5:**
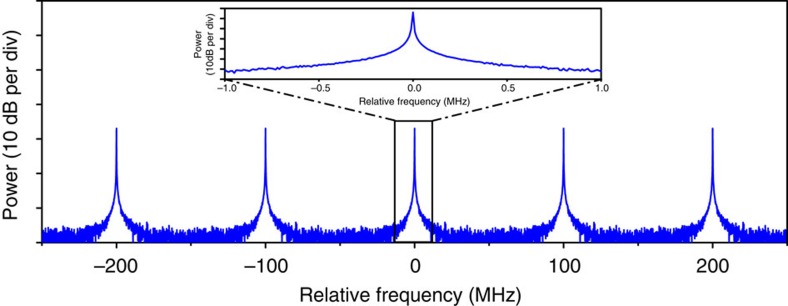
A simulated high-resolution spectrum of the silicon wire output. The spectrum of the supercontinuum frequency comb is simulated over a 500-MHz bandwidth in the vicinity of 1,586 nm (198 THz). It reveals comb lines separated by 100 MHz in the. A high-resolution (10 kHz) inset around a comb line is also shown.

To emphasize the comb structure of the supercontinuum spectrum, which results from the pulse-to-pulse coherence, the spectrum of the pulse train at the output of the chip was simulated with a resolution of 10 kHz in a narrow band interval. The spectrum is simulated by first generating a set of pulses including the input shot noise, but excluding timing jitter and residual intensity noise, as discussed above. These pulses were stacked together in a pulse train with a repetition frequency of 100 MHz. The Fourier transform was calculated to generate the spectrum of the pulse train (see Supplementary Methods and Supplementary Fig. 3 for details). Figure 5 shows the spectrum of a train of 1,000 pulses, calculated in a 500-MHz interval at 1,586 nm. The independent comb lines can clearly be seen. The inset of Fig. 5 shows one individual comb line sampled with a resolution of 10 kHz by calculating the spectrum of a pulse train consisting of 10,000 pulses. Similar simulations were performed in an interval at 2,418 and 2,580 nm, confirming the comb structure of the supercontinuum (see Supplementary Fig. 4). In the simulations, the width of the comb lines is only limited by the time window used.

Interestingly, such nonlinear processes in silicon wires can be harnessed with other mid-infrared ultrashort pulse pump laser systems of different wavelength than the OPO employed here. For example, our simulations (Supplementary Discussion and Supplementary Fig. 5) indicate that a thulium-doped mode-locked fibre laser can be used as well to generate a phase-coherent octave-spanning supercontinuum in a dispersion-engineered silicon waveguide.

## Discussion

Using a silicon nanowire on a chip, we have demonstrated an octave-spanning frequency comb spanning from the telecom wavelength window ~1,500 nm to the mid-infared wavelength range at 3,300 nm. Such frequency comb is readily suitable for direct frequency comb spectroscopy, particularly for dual-comb spectroscopy with, for example, adaptive sampling^25^. Improved dispersion engineering could potentially extend the supercontinuum over the whole transparency window of the SOI platform (1,100–4,000 nm), limited by the buried oxide. Even broader bandwidths could be further obtained up to 5,500 nm with silicon on sapphire waveguide platforms^31^,^32^. By using waveguide designs where the buried oxide is removed^14^,^15^,^33^, the entire silicon transparency window (up to 8,500 nm) could be covered. As many molecules have strong rovibrational lines in the mid-infrared range, such developments would contribute in expanding the intriguing capabilities of molecular spectroscopy with frequency combs to the molecular fingerprint region. Such broadband supercontinua may also lead to self-referenced mid-infrared frequency comb systems, as needed for precision measurements in frequency metrology and in some implementations of direct frequency comb spectroscopy^1^. The rapid progress in the development of miniaturized mid-infrared frequency comb generators, as reported for instance with quantum cascade lasers^2^,^4^ or high-quality factor microresonators^34^, might lead to an entirely new strategy for a compact source of ultrashort pulses in the future. Our work would then represent an essential building block paving the road for an octave-spanning frequency comb entirely generated on a chip. Such prospect would be of interest to, for example, chemical sensing, calibration of astronomical spectrographs, environmental monitoring or free-space communications.

## Methods

### Description of the mid-infrared frequency comb seed source

The frequency comb generator that seeds the silicon waveguide is a home-made femtosecond synchronously pumped OPO. Its design and characterization are described in ref. 19. Here we just reproduce the details that are useful for the description of the present experiment. The pump source of the OPO is a Kerr-lens mode-locked Ti:sapphire oscillator with a repetition frequency of 100 MHz, an average power of 1 W, a central wavelength of 790 nm and a pulse duration of 20 fs. The nonlinear crystal of the OPO is made of MgO:PPLN with a fan-out grating interaction length of *l*=500 μm. The OPO cavity is a dispersion-controlled four-mirror standing-wave design with two planoconcave mirrors and four plane mirrors. We tune the central wavelength of the idler of the OPO to 2,290 nm. The average output power is 35 mW. The idler spectrum is shown in Fig. 2b. We measure the pulse duration with a home-made autocorrelator based on two-photon absorption in a InGaAs photodetector. The autocorrelation (Supplementary Fig. 1) reveals a pulse duration of 72 fs, assuming a *sech*^2^ profile.

### Simulations

The spectral evolution of the pulses along the waveguide is simulated by solving the generalized nonlinear Schrodinger equation numerically using a split-step approach^35^. We solve


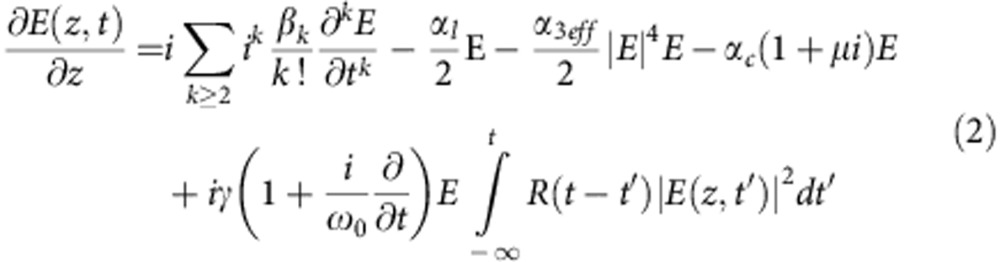


Here *E*(*z*, *t*) is the envelope of the electric field of the short pulses, *β*_k_ is the kth order dispersion coefficient, *α*_l_ the linear propagation loss, *α*_3eff_ the effective third-order absorption coefficient, *α*_c_ the free carrier absorption coefficient, *μ* takes the free carrier dispersion in account, *γ* is the nonlinear parameter of the waveguide, while the integral takes in account the fractional Raman response. The effective third-order absorption coefficient can be calculated as 
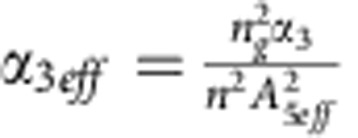
 (ref. 36), where *α*_*3*_ is the third-order nonlinear absorption coefficient in silicon of ~2.5 × 10^−26 ^m^3 ^GW^−2^ (refs 27, 37) and *A*_5eff_=0.5 μm^2^ the fifth-order mode area. The carrier-induced absorption coefficient is proportional to the carrier density *N*_c_, such that *α*_c_=*σN*_c_, where *σ*=2.77 × 10^−21 ^m^2^ (ref. 38), while 
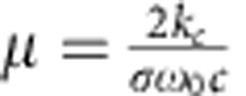
with *k*_c_=1.35 × 10^−27 ^m^3^ (ref. 38). The evolution of the carrier density itself can be calculated as 
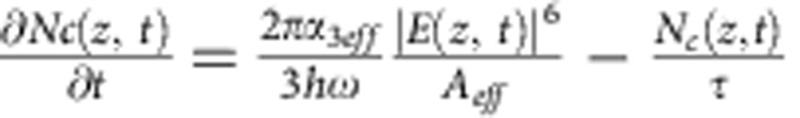
 (ref. 38), where *h* is Planck’s constant and *τ* the carrier lifetime, estimated to be 1 ns (ref. 39). It was assumed that the pulse was a hyperbolic secant with a full-width at half-maximum of 70 fs.

## Author contributions

B.K. performed the numerical dispersion design calculations with guidance from R.B. and G.R., J.V.C. and P.V. supervised the waveguide device fabrication process. B.K., T.I., S.H. and M.Y. performed the supercontinuum generation experiment as well as the beat note experiment with guidance and supervision from T.W.H. and N.P. T.I., S.H. and M.Y. performed the autocorrelation experiment under the supervision of N.P. B.K., F.L. and S.C. performed the simulations on the coherence. B.K. drafted the manuscript. All authors provided comments and suggestions for improvements.

## Additional information

**How to cite this article:** Kuyken, B. *et al*. An octave-spanning mid-infrared frequency comb generated in a silicon nanophotonic wire waveguide. *Nat. Commun.* 6:6310 doi: 10.1038/ncomms7310 (2015).

## Supplementary Material

Supplementary Information Supplementary Figures 1-5, Supplementary Methods, Supplementary Discussion and Supplementary References
