# Supplementary material for: An octave-spanning mid-infrared frequency comb generated in a silicon nanophotonic wire waveguide
Source: Nat Commun. 2015 Feb 20;6:6310. doi: 10.1038/ncomms7310 (PMC4346629; doi:10.1038/ncomms7310)
Supplement: Supplementary Information — Supplementary Figures 1-5, Supplementary Methods, Supplementary Discussion and Supplementary References [file ncomms7310-s1.pdf]

Supplementary Figures

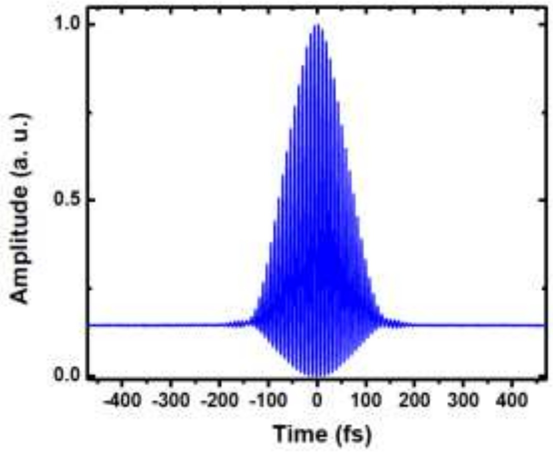

Supplementary Figure 1: Interferometric autocorrelation of the pulse at 2,290 nm. The idler of the optical parametric oscillator is used as a seed source for the silicon waveguide.

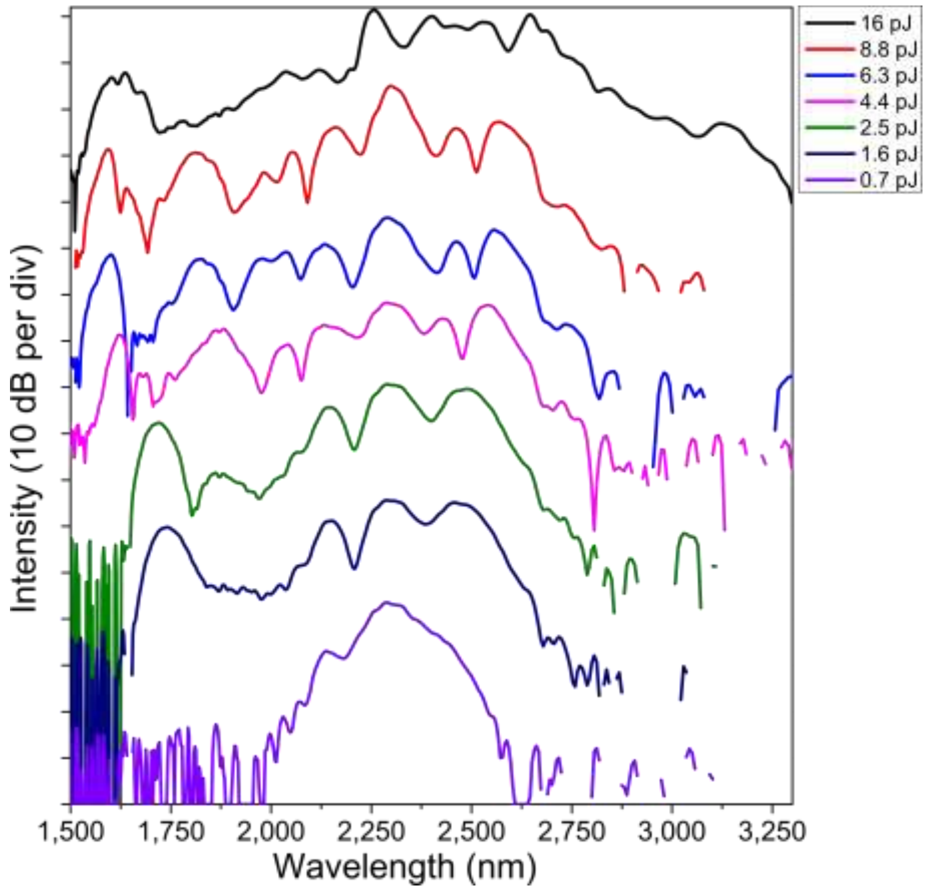

Supplementary Figure 2: Experimental spectra of the silicon chip output as a function of the input pulse energy

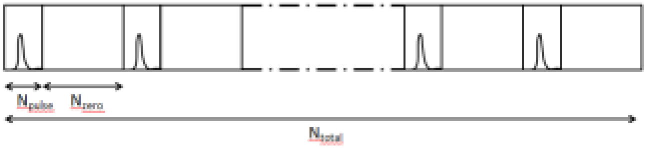

Supplementary Figure 3: A pulse train consisting of a number of pulses sampled with N_{pulse} samples.

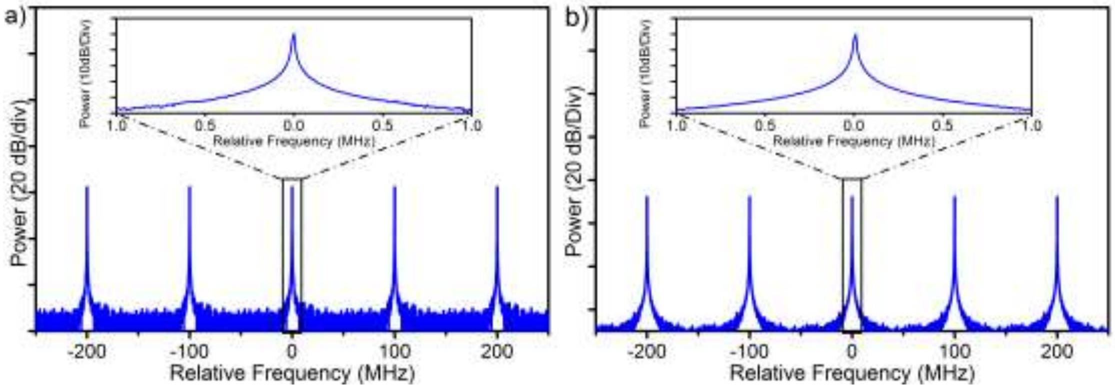

Supplementary Figure 4: A high resolution spectrum of the comb. A spectrum simulated in the vicinity of a) 2,418 nm (124 THz) and b) 2,580 nm (116 THz) . The spectra are sampled at 100 kHz over a 500-MHz bandwidth, revealing the comb lines separated by 100MHz in the supercontinuum frequency comb. The insets show a high resolution (10 kHz) plot around a comb line.

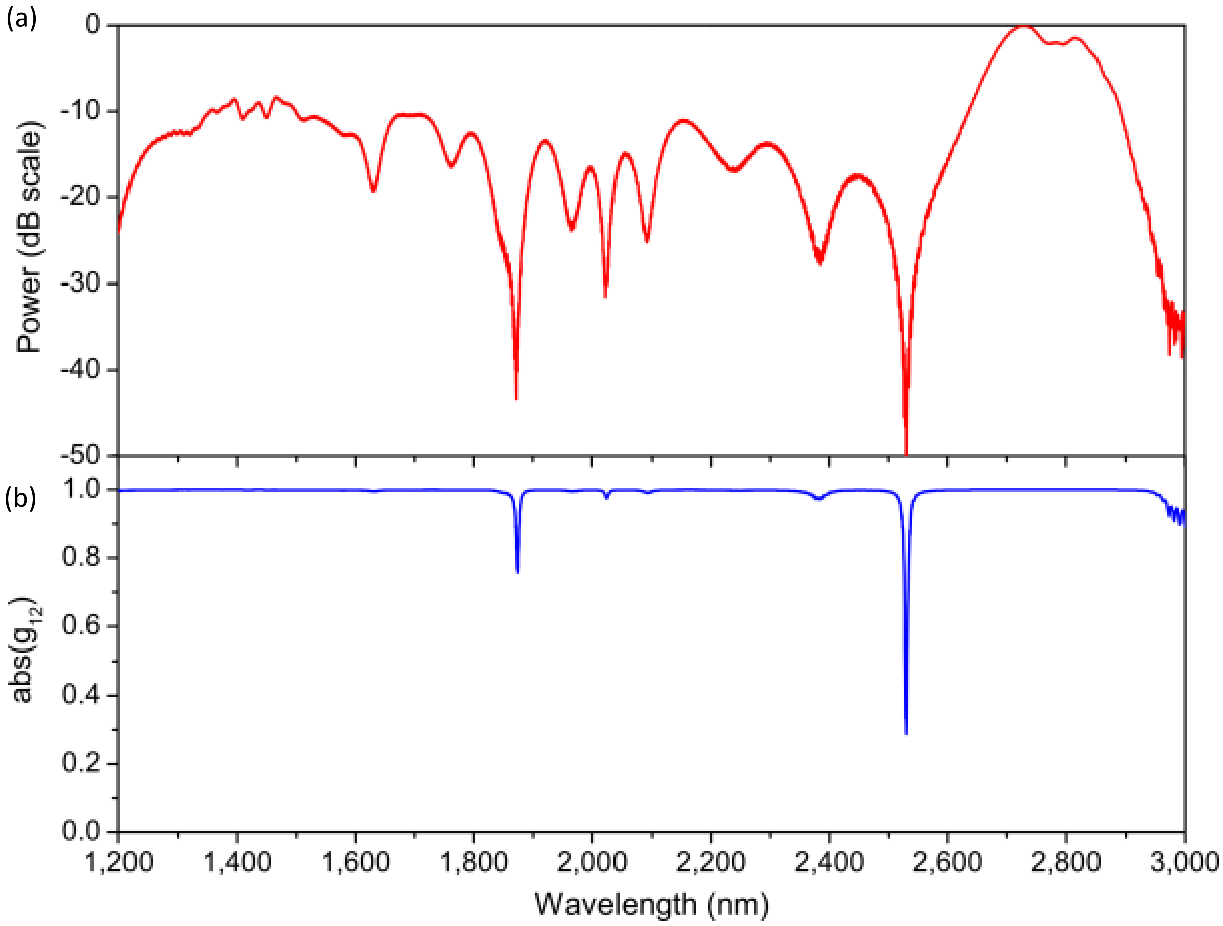

Supplementary Figure 5: Supercontinuum simulations under thulium-doped fiber femtosecond laser pumping. a) The octave-spanning spectrum at the output of a 4-mm silicon waveguide and b) the coherence of the spectrum as a function of the wavelength.

Supplementary Methods

Detailed experimental setup and alignment procedure

The experimental setup consisted of two x,y,z stages (Newport 562 Ultralign) which could hold a lensed fiber (OZ optics, lensed fiber with a 2 µm spot size) or a chalcogenide lens (Thorlabs C037TME with a focal length of 1.87 mm and a numerical aperture NA=0.85) to couple in and out of the silicon chip. The silicon chip was held on a x,y stage. The major difficulty of working in the mid-infrared is the visualization of the mid-infrared light. Mid-infrared cameras remain costly and heat sensitive paper used to visualize mid-IR beams lack the sensitivity needed. To align the mid-infrared light with the chalcogenide lenses several steps where undertaken. First, the chip and the lensed fibers were imaged with a 40x high-NA long-working-distance objective on a CMOS camera. Laser light with a wavelength of 1,064 nm was coupled to the lensed fibers. With the help of the CMOS camera, sensitive to the laser light, the lensed fibers were aligned with the waveguides. Next, a 1,550 nm source was coupled to one of the lensed fibers and the transmission was optimized with the help of a power meter. Then the lensed fiber, collecting the output of the chip was removed and the chalcogenide lens was placed on the x,y,z stage. A pyroelectric camera (Ophir optics, Pyrocam III) was used to visualize the beam at 1,550 nm imaged by the chalcogenide lens. The position of the lens was moved such that the output beam becomes collimated. When the collimated beam was visualized on the camera, a mid-IR (about 2,400 nm) continuous wave light source was coupled to the lensed fiber. With the help of the camera, the output beam (altered due to the dispersion of the lens) was again collimated by changing the position of the lens with respect to the chip. In a next stage, the 2,400 nm output beam was spatially overlapped with the output of the femtosecond OPO (also tuned at 2,400 nm). The lensed fiber was connected to a mid-IR photodiode. With the power as a feedback signal, the lens position and the coupling are optimized. In a last step, the second lensed fiber was replaced with a lens and the output beam collimated with the help of the mid-IR camera. To increase the coupling to the chip, the output of the femtosecond OPO seed source was sent to a telescope having two CaF_2 lenses to reduce the diameter of its beam by a factor of two (about 4mm). Furthermore a half-wave plate was used to change the vertically polarized (TM) output light of the femtosecond OPO to a horizontal polarization (TE).

The simulation of the frequency comb with a high resolution.

To simulate the frequency comb with a high resolution (10kHz), the Fourier transform of a pulse train was calculated. First, 10,000 individual pulses were simulated assuming shot noise on the input pulse. The simulation of these pulses is identical to the method used for calculating the first order coherence function $g_{12}(\omega)$. A temporal simulation window of 35 ps consisting of 8192 samples (N_{pulse} samples, see Figure S3), a resolution of 4.27 fs, was assumed when analytically solving the generalized nonlinear Schrödinger equation. To construct the pulse train, it is assumed that the amplitude of the pulse train is zero in between pulses. Under this assumption, the Fourier transform of the very long pulsetrain can be calculated more efficiently.

The discrete Fourier transform of the one dimensional array $f(t_n)$ representing the pulse train sampled at (N_{freq} points) is as follows:

$$F(k) = \sum_n f(t_n)\exp(-j\frac{2\pi kn}{N_{freq}})(1)$$

Here, $f(t_n)$ is the complex amplitude of the pulse train at $t_n = 4.3fs \times n$ and N is the total number of elements in the pulse train. We define N_{pulse}=8192 the number of samples representing one temporal pulse and N_{zero} the amount of zeros such that two pulses are separated by 10 ns (a repetition rate of 100 MHz is assumed) N_{zero}= 2332379. Furthermore we name $f_m(t_n)$ the mth pulse in the pulse train such that $f_m(t_n) = f(t_{mN+n})$. The discrete Fourier transform (1), sampled at a resolution of N_{freq} points of the pulse train becomes:

$$F(k) = \sum_n f_0(t_n)\exp\left(-j\frac{2\pi kn}{N_{freq}}\right)$$

$$+\sum_n f_1(t_n)\exp\left(-j\frac{2\pi k(n+N_{pulse}+N_{zero})}{N_{freq}}\right) + \sum_n f_2(t_n)\exp\left(-j\frac{2\pi k(n+2(N_{pulse}+N_{zero}))}{N_{freq}}\right) + \cdots \; (2)$$

The expression (2) can be simplified as follows:

$$F(k) = \sum_m \exp(-j\frac{2\pi k(N_{pulse}+N_{zero})}{N_{freq}})\sum_n f_m(t_n)\exp\left(-j\frac{2\pi kn}{N_{freq}}\right) \; (3)$$

such that, for each frequency, significantly less numerical operations have to be performed. Figure 5 shows a high resolution spectrum in the vicinity of 1,586 nm calculated with this approach. Figure S4 shows a part of the same high resolution spectrum at 2,418 nm and 2,580 nm. As can be seen in Fig.5 and Fig. S4, the high resolution spectrum is clearly a frequency comb, confirming that the supercontinuum process preserves the coherence.

Supplementary Discussion

Simulation of a phase-coherent supercontinuum by pumping a silicon waveguide with a mode-locked thulium-doped fiber laser.

A thulium-doped fiber mode-locked laser can be used as an alternative to an OPO. These laser systems tend to operate at 2,000 nm [1]. Here, we simulate the output spectrum of a short dispersion engineered silicon photonic wire waveguide. The silicon photonic waveguide in the simulation is 900 nm wide and 220 nm high and has an air cladding. The whole dispersion profile can be found in Figure 1 of [2]. The second, third, fourth and fifth dispersion coefficients are found to be -0.25676 $ps^2.m^{-1}$, 3.3945x10^{-3} $ps^3.m^{-1}$, -4.8142x10^{-5} $ps^4.m^{-1}$, -2.9079x10^{-7} $ps^5.m^{-1}$, 1.3884x10^{-8} $ps.m^{-1}$ at the pump wavelength. The nonlinear parameter is simulated to be 282+40i $(W.m)^{-1}$. It is assumed that the pulses produced by the thulium mode-locked laser have a pulse duration of 65 fs and are centered at 2,000 nm [1]. The silicon photonic wire is assumed to be 4 mm long and to have a loss of

2 dB.cm^{-1}. As shown in Supplementary Figure 5, the spectrum of the pulses at the output of the silicon waveguide is octave-spanning when the coupled peak power in the silicon waveguides is 50 W. The supercontinuum is found to be coherent over its full span except at a narrowband at 1,880 nm and 2,560 nm where the spectral density of the supercontinuum is very weak.

Supplementary references:

[1] Granzow, N. et al. Mid-infrared supercontinuum generation in As_2S_3-silica "nano-spike" step-index waveguide. Opt. Express **21**, 10969-10977 (2013).

[2] Kuyken, B. et al. Mid-infrared to telecom-band supercontinuum generation in highly nonlinear silicon-on-insulator wire waveguides. Opt. Express **19**, 20172-20181 (2011).
